# Supplementary material for: High crosstalk suppression in InGaAs/InP single-photon avalanche diode arrays by carrier extraction structure
Source: Nat Commun. 2024 Jan 18;15:593. doi: 10.1038/s41467-023-43341-9 (PMC10796904; doi:10.1038/s41467-023-43341-9)
Supplement: Supplementary file 1 — Supplementary Information [file 41467_2023_43341_MOESM1_ESM.pdf]

Supplementary Information

High crosstalk suppression in InGaAs/InP single-photon avalanche diode arrays by carrier extraction structure

Yongsheng Tang[1,2], Rui Wang[1], Xiaohong Yang[1,3*]✉, Tingting He[1,3], Yijun Liu[1,3], Meng Zhao[1]

[1]State Key Laboratory of Integrated Optoelectronics, Institute of Semiconductors, Chinese Academy of Sciences, Beijing 100083, China.

[2]School of Electronic, Electrical and Communication Engineering, University of Chinese Academy of Sciences, Beijing 100049, China.

[3]College of Materials Science and Opto-Electronic Technology, University of Chinese Academy of Sciences, Beijing 100049, China.

Correspondence: Xiaohong Yang (✉e-mail: xhyang@semi.ac.cn)

Table of contents of supplementary information

Supplementary Fig. 1 | The distribution of the electric field and valence band in the device at 1 V excess bias voltage (V_{ex} = 1 V).

Supplementary Fig. 2 | Photocurrent responses and gain voltage characteristics when the light absorption is located at different positions.

Supplementary Fig. 3 | SEM images of diffusion profiles for the pixel and the carrier extraction structure (CES).

Supplementary Fig. 4 | The distribution of the electric field near the unity gain point (punch-through voltage, V_{ph}).

Supplementary Fig. 5 | The distribution of the lateral electric field (E_r) and the valence band between the pixel anode and carrier extraction structure (CES), when the distance between the pixel and CES is 12 µm.

Supplementary Fig. 6 | The influence of the pixel bias voltage on carrier extraction structure (CES).

Supplementary Fig. 7 | DC crosstalk measurement setups.

Supplementary Fig. 8 | The impact of the floating guard ring (FGR) on the crosstalk.

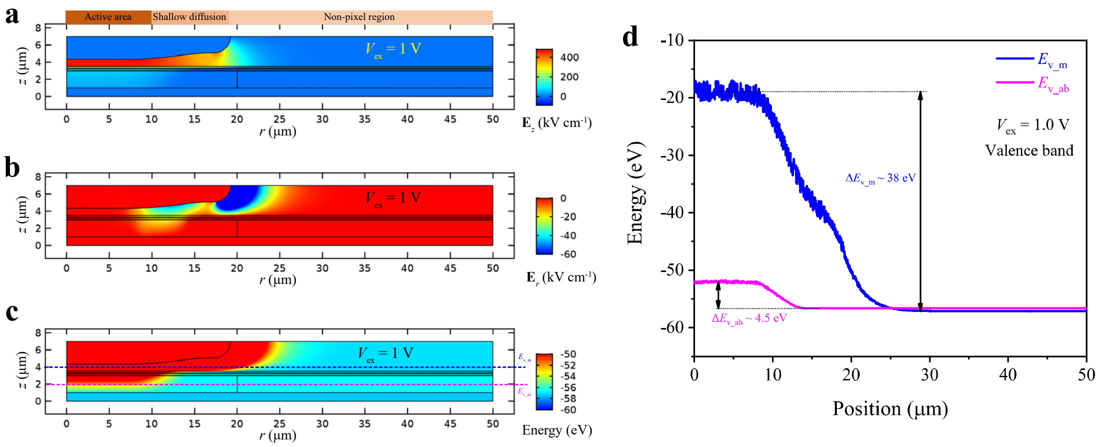

Supplementary Fig. 1 | The distribution of the electric field and valence band in the device at 1 V excess bias voltage (V_{ex} = 1 V). The distribution of the electric field **a** in the vertical direction ($\mathbf{E}_z$); and **b** in the horizontal electric field component ($\mathbf{E}_r$). The valence band distribution **c** in the device cross-section; and **d** in the middle of the InP multiplication layer (E_{v_m}, blue dotted line in Supplementary Fig. 1c) and the InGaAs absorption layer (E_{v_ab}, pink dotted line in Supplementary Fig. 1c). The InP cap layer below the shallow diffusion zone is also depleted, with a strong vertical electric field (*z*-direction), as shown in Supplementary Fig. 1a. The horizontal electric field mainly distributes at the edges of the active area and the shallow diffusion region; and importantly, the non-pixel region is a neutral region (the horizontal electric field is 0 kV cm^{-1}), as shown in Supplementary Fig. 1b. Therefore, the avalanche hot carriers diffused outside the multiplication region will be recaptured through the shallow diffusion zone. On the other hand, there is also a large potential barrier ΔE_v between the active area and non-pixel region, which blocks the diffusion of minority carriers from the active area into adjacent pixels, so the contribution of direct electrical crosstalk is negligible, as shown in Supplementary Fig .1c,d. (For the InP layer and the InGaAs absorption layer, the minority carriers are holes, so we only analyzed the distribution of the valence band.) However, for the photogenerated holes in the non-pixel region, they can be easily collected by the active area, thus leading to the optical-electrical crosstalk.

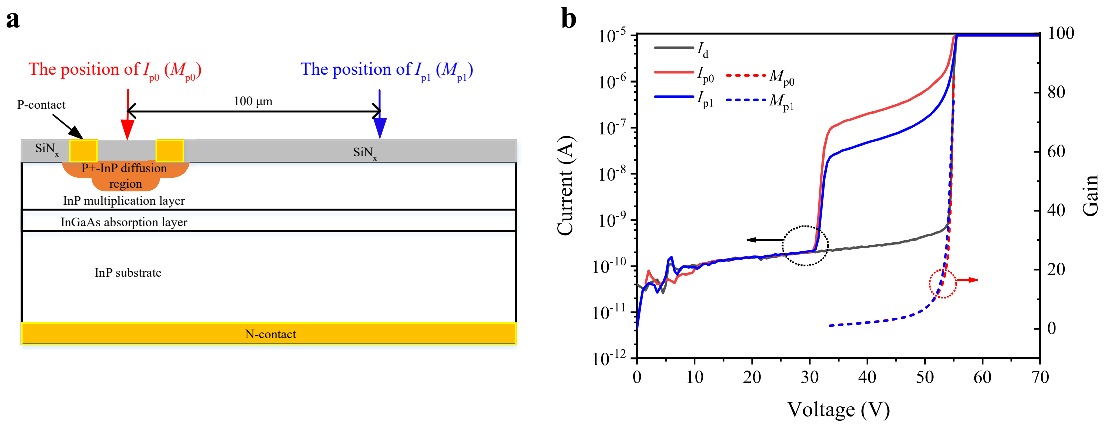

Supplementary Fig. 2 | Photocurrent responses and gain voltage characteristics when the light absorption is located at different positions. a The schematic diagram of light absorption at different positions, and **b** the measured current-voltage (I-V) characteristics and voltage dependence of the multiplication factor (M-V) of SPAD. I_{p0} and M_{p0} represent the light absorption located at the center of the active area, and I_{p1} and M_{p1} represent the light absorption located at 100 µm outside the center of the active area. The wavelength of light is 1550 nm. A front-illuminated SPAD with a 20 µm active area diameter was used. The device epitaxial structures are the same as the pixel in arrays. Photocurrent gains M_{p0} and M_{p1} are almost the same, which increase to 85 and 78 respectively before the breakdown, illustrating that the photogenerated holes in the InGaAs layer of the non-pixel region could also be captured by the InP multiplication layer to provide Geiger-mode (GM) avalanche gain. I_{p1} is large and has the same order of magnitude as I_{p0}, nearly 25% (I_{p1}/I_{p0}) of them (some photogenerated holes recombined as they diffuse from the non-pixel region to the active area) would be captured by InP multiplication region. The collection efficiency is high; and as the light absorption position is closer to the pixel, the collection efficiency would be higher. Therefore, the optical-electrical crosstalk is also an important source of crosstalk in planar InGaAs/InP single-photon avalanche diode (SPAD) arrays. And the results can also verify the efficient collection efficiency of our carrier extraction structure (CES) for photogenerated crosstalk holes in the non-pixel region.

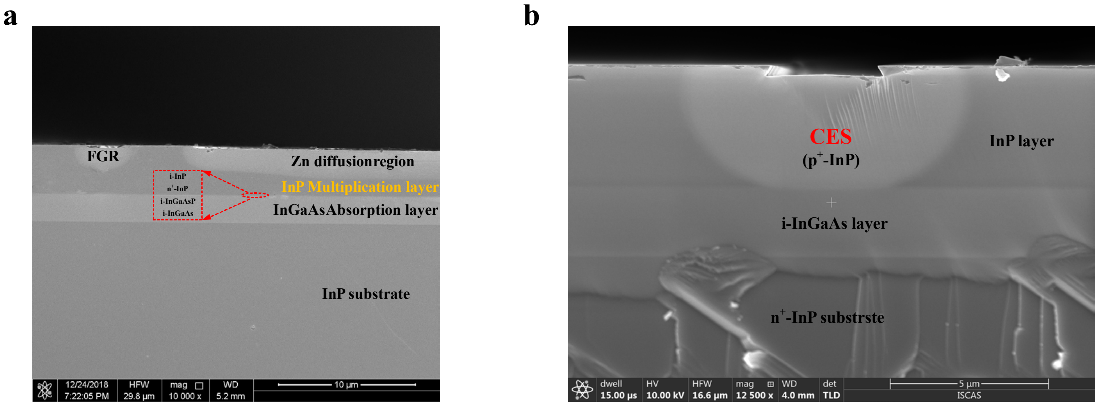

Supplementary Fig. 3 | SEM images of diffusion profiles for the pixel and the carrier extraction structure (CES). a The typical planar InGaAs/InP SPAD cross-section, with layers structure, double diffusion profile, and floating guard ring (FGR). **b** The CES cross-section. The CES is a p^+-doped channel, which is similar to forming a PIN detector in the non-pixel region. Therefore, the CES can collect the crosstalk photogenerated holes in the non-pixel region to achieve crosstalk suppression. The obvious lateral diffusion can be seen from the SEM images, which results in wider p-doped regions than designed.

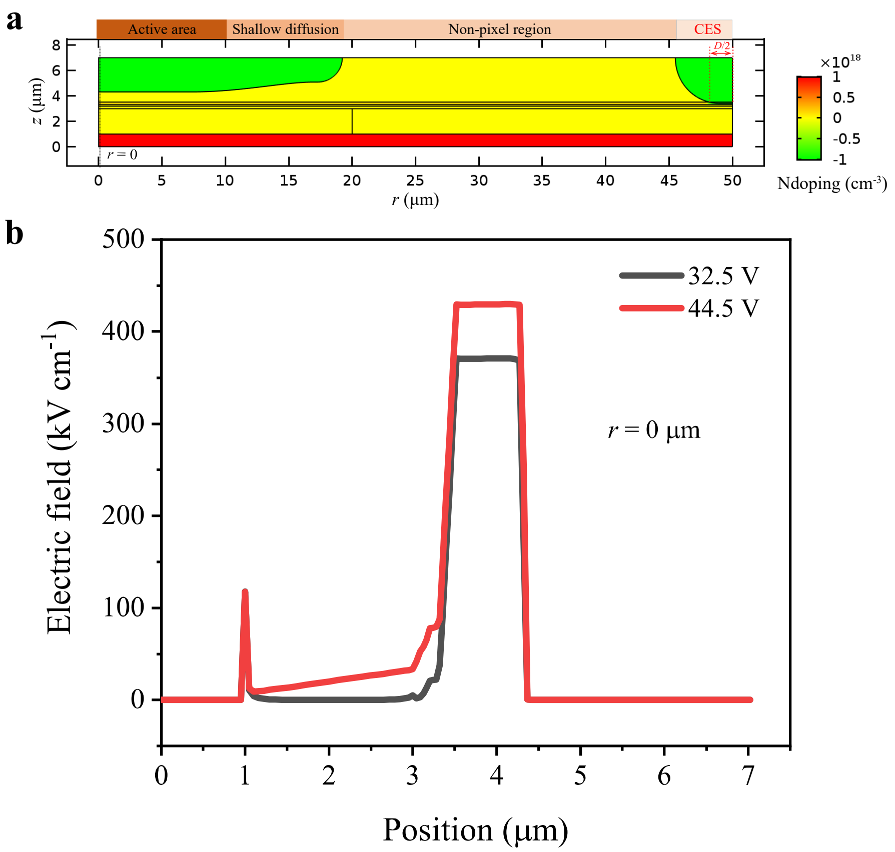

Supplementary Fig. 4 | The distribution of the electric field near the unity gain point (punch-through voltage, V_{ph}), at r = 0 μm. a A 2-D rotationally symmetric model in COMSOL for simulation analysis. **b** The distribution of the electric field at the center of single-photon avalanche diode (SPAD) (r = 0 μm, as shown in Supplementary Fig. 4a) near the unity gain point (V_{ph}), the figure shows the electric field component in the z-direction. At unity gain (32.5 V), the charge layer is depleted, but the electric field in the InGaAs absorption layer is still 0 kV cm^{-1}. When the pixel reverse bias voltage increases to 44.5 V, there is a non-zero electric field in the InGaAs layer, which achieves electrical punch-through. So, the photogenerated holes in the active area are completely collected by the pixel above 44.5 V.

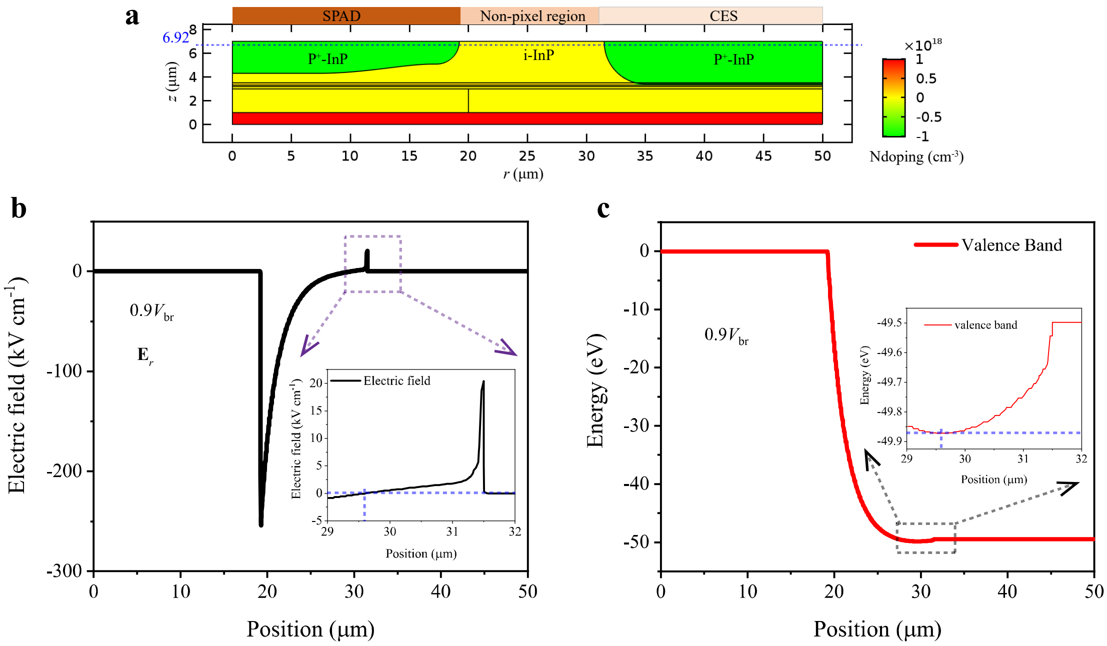

Supplementary Fig. 5 | The distribution of the lateral electric field (E$_r$) and the valence band between the pixel anode and carrier extraction structure (CES), when the distance *d* between the pixel and CES is 12 μm. a A 2-D rotationally symmetric model in COMSOL for simulation analysis. The distribution of **b** lateral electric field (**E**$_r$) and **c** the valence band between the pixel anode and the CES at the position of 100 nm below the surface (light blue dotted line in Supplementary Fig. 5a), when the pixel is biased at $0.9V_{br}$ and $V_{ces} = 0$ V. A p-i-p structure forms between the pixel anode and the CES, as shown in Supplementary Fig. 5a. When the distance between the pixel anode and CES is close, the i-InP is depleted, but due to the p-i-p structure, there is a back-to-back **E**$_r$ in the depletion region, as shown in Supplementary Fig. 5b. Therefore, a potential barrier generates between them, as shown in Supplementary Fig. 5c. When the pixel is biased at $0.9V_{br}$ and $V_{ces} = 0$ V, this potential barrier is 0.37 eV. The holes from CES can cross this low potential barrier and flow to the pixel anode, which leads to a hole leakage current between the pixel anode and CES.

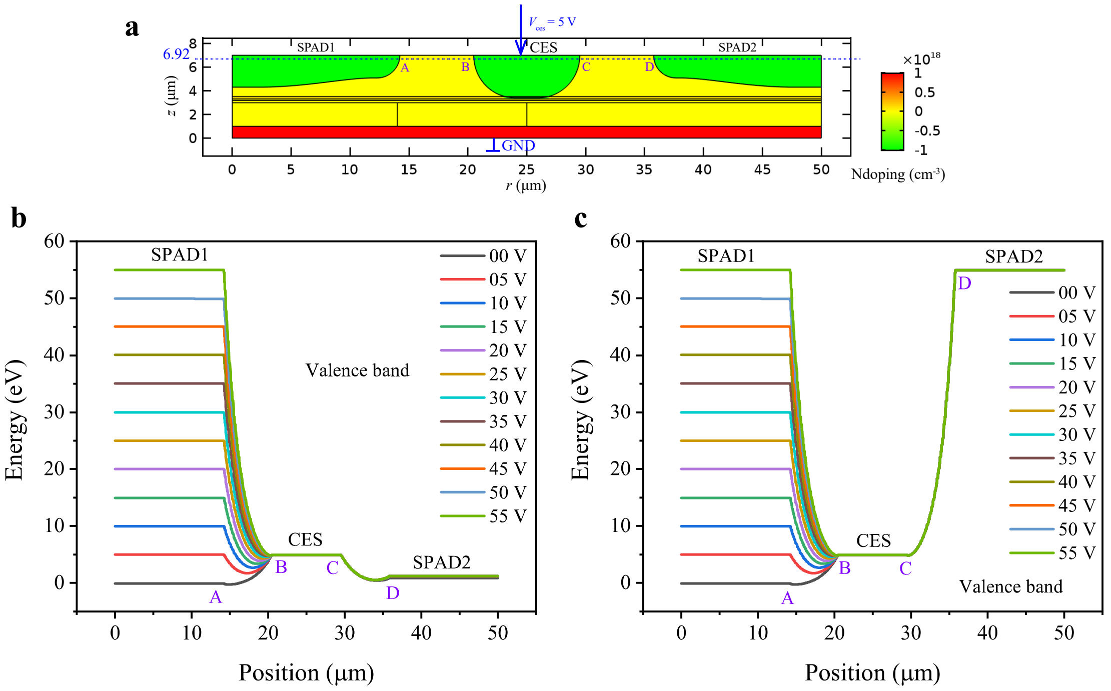

Supplementary Fig. 6 | The influence of the pixel bias voltage on the carrier extraction structure (CES). a A 2-D model of the nearest neighbor pixels is used for simulation analysis in the array with a 50 μm pixel pitch and the distance between the pixel and CES (*d*) is ~ 6 μm. The active area diameter of the pixel is 10 μm. And the reverse bias voltage applied on the CES (V_{ces}) is 5 V. The distribution of the valence band at the position of 100 nm below the surface (light blue dotted line in Supplementary Fig. 6a) at bias voltage of single-photon avalanche diode 1 (SPAD1) from 0 V to 55 V, **b** when SPAD2 is OFF (SPAD2 does not work), **c** when SPAD2 is kept ON and operates at 55 V (V_{br}). The bias voltage of SPAD1 only affects the distribution of the valence band between A and B, it does not affect the valence band distribution and the potential barrier between C and D. And the working state of SPAD2 does not affect the potential barrier between A and B. Therefore, it is effective to investigate the working state or crosstalk suppression effect of the array by simulating and measuring the characteristics of a single pixel in the array (ignoring the effects of uniformity).

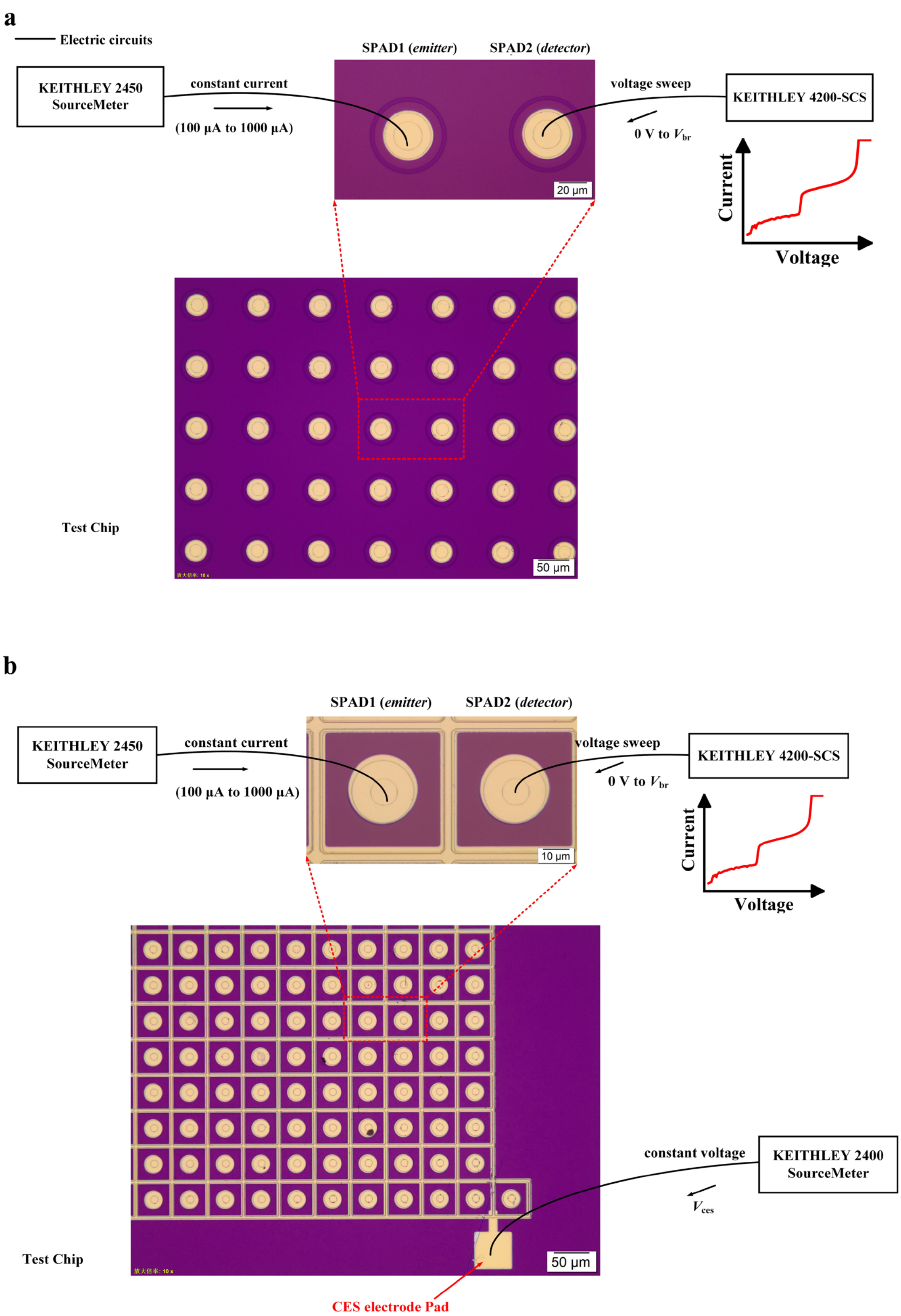

Supplementary Fig. 7 | DC crosstalk measurements setups. The micrographs show the anode surface of a local array. The carrier extraction structure (CES) and pixels share a common N electrode on the bottom. Crosstalk measurements setups for the array **a** without CES and **b** with CES.

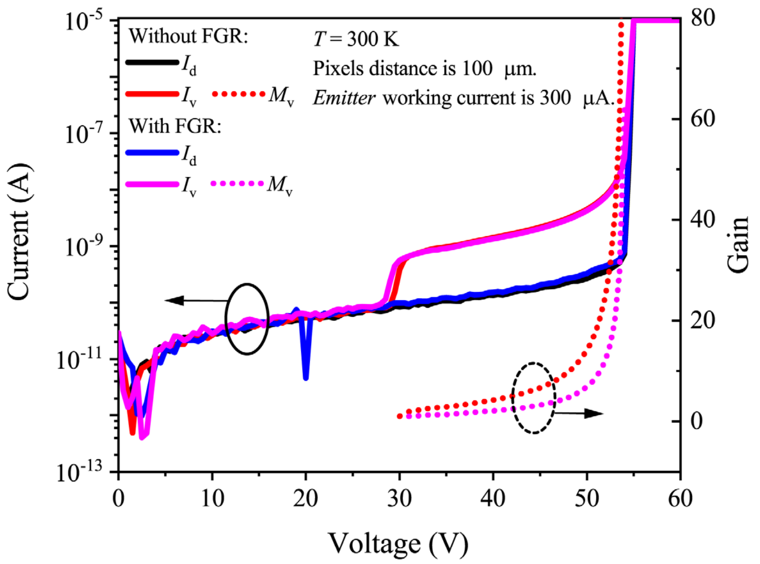

Supplementary Fig. 8 | The impact of the floating guard ring (FGR) on the crosstalk. DC crosstalk measurement results between the nearest neighbor pixels at a temperature (T) of 300 K. The solid lines represent the measured current-voltage (I-V) characteristics and the dashed lines represent the voltage dependence of the gain (M-V). Measured results show that the FGR has almost no suppression effect on the crosstalk.
